# Cytogenomic Characterization of Transposable Elements and Satellite DNA in *Passiflora* L. Species

**DOI:** 10.3390/genes15040418

**Published:** 2024-03-27

**Authors:** Gonçalo Santos Silva, Margarete Magalhães Souza, Vanessa de Carvalho Cayres Pamponét, Fabienne Micheli, Cláusio Antônio Ferreira de Melo, Sárah Gomes de Oliveira, Eduardo Almeida Costa

**Affiliations:** 1 Laboratório de Melhoramento de Plantas, Departamento de Ciências Biológicas, Universidade Estadual de Santa Cruz (UESC), Ilhéus 45662-900, BA, Brazil; goncaloss21@hotmail.com (G.S.S.); van_cgc@yahoo.com.br (V.d.C.C.P.); fabienne.michele@cirad.fr (F.M.); clausiomelo@gmail.com (C.A.F.d.M.); educa@gmail.com (E.A.C.); 2 CIRAD, UMR AGAP, F-34398 Montpellier, France; 3 Departamento de Botânica, Instituto de Biociências, Universidade de São Paulo (USP), São Paulo 01049-010, SP, Brazil; sarahg.oliveira@gmail.com

**Keywords:** FISH, *Passiflora alata*, *Passiflora cincinnata*, *Passiflora edulis*, satellite DNA, transposable elements, Ty1/Copia, Ty3/Gypsy

## Abstract

The species *Passiflora alata*, *P. cincinnata*, and *P. edulis* have great economic value due to the use of their fruits for human consumption. In this study, we compared the repetitive genome fractions of these three species. The compositions of the repetitive DNA of these three species’ genomes were analyzed using clustering and identification of the repetitive sequences with RepeatExplorer. It was found that repetitive DNA content represents 74.70%, 66.86%, and 62.24% of the genome of *P. alata*, *P. edulis*, and *P. cincinnata*, respectively. LTR Ty3/Gypsy retrotransposons represent the highest genome proportions in *P. alata* and *P. edulis*, while Ty1/Copia comprises the largest proportion of *P. cincinnata* genome. Chromosomal mapping by Fluorescent In Situ Hybridization (FISH) showed that LTR retrotransposons have a dispersed distribution along chromosomes. The subtelomeric region of chromosomes is where 145 bp satellite DNA is located, suggesting that these elements may play important roles in genome structure and organization in these species. In this work, we obtained the first global characterization of the composition of repetitive DNA in *Passiflora*, showing that an increase in genome size is related to an increase in repetitive DNA, which represents an important evolutionary route for these species.

## 1. Introduction

The genus *Passiflora* L. comprises species known as passion fruit, which are distributed across the tropics and subtropics [1]. Many passion fruit species produce edible fruits, which can be consumed in natura in the form of juices or in various culinary dishes, in addition to their uses for medicinal and ornamental purposes [2,3]. Currently, based on morphological data, the genus *Passiflora* is divided into four subgenera: *Astrophea* (DC.) Mast., *Decaloba* (DC.) Rchb., *Deidamioides* (Harms) Killip, and *Passiflora* [4]. In addition, its molecular phylogeny fully or partially corroborates this classification [4,5,6,7,8].

The species of greatest economic interest belong to the subgenus *Passiflora*, section *Passiflora* [2], with *P. alata* Curtis, *P. cincinnata* Mast., and *P. edulis* Sims being the most economically important for fruit production in Brazil [9]. However, *P. edulis* stands out due to its large fruit production, occupying more than 95% of the passion fruit trees planted in the country [10] and producing 683,993 tons of fruit in 2021 [11]. Considering the diversity of wild passion fruit in Brazil and its economic value, it represents a potential area for exploration and a promising research field in plant breeding.

The amplification process of repetitive DNA has not yet been fully understood in plan species. However, it has been demonstrated that plants with large genomes contain high proportions of repetitive DNA, especially transposable elements and satellite DNAs (satDNAs) [12,13]. Recently, genome sequencing has been revolutionized by next-generation technologies and new bioinformatics tools [14,15], making it possible to perform the global characterization of repetitive DNA in several plant genomes as well as conduct comparative analyses within or between genomes [12,16,17,18,19,20,21]. In *P. edulis*, previous studies analyzed repetitive DNA from 10,000 sequences inserted into BACs (Bacterial Artificial Chromosomes) [22] and 11,493,782 paired-end reads using RepeatExplorer [23]. It was observed that repetitive DNA corresponded to 19.6% and 87% of the sequences, respectively. Considering that in plants, the evolution of repetitive DNA occurs faster than gene sequence, geneticists and plant breeders can use this type of DNA as a molecular marker to map important genes, analyze genetic diversity, and study the process of speciation and genome evolution [17].

The genome size of *Passiflora* subgenus varies from 0.26 to 2.68 picograms (1C) between the species [24,25,26]. Since species in the *Passiflora* subgenus have the same chromosomal number, 2*n* = 18, increases in genome size are suggested to be related to increases in repetitive DNA [27].

In this context, the objective of this work was to verify the composition of repetitive DNA in *P. alata*, *P. cincinnata*, and *P. edulis*. A comparative analysis of repetitive DNA was performed among these species to identify the main groups of repetitive DNA of which the genomes of these species are composed and verify their distribution across the chromosomes.

## 2. Materials and Methods

### 2.1. Plant Material

Samples of *Passiflora alata* (2*n* = 18), *P. cincinnata* (2*n* = 18), and *P. edulis* (2*n* = 18) were kept in the working collection of Passifloras at Universidade Estadual de Santa Cruz (UESC), located in the municipality of Ilhéus, Bahia, Brazil (39 10′ W, 14 39′ S, 78 m above sea level). *P. alata* was donated by the Universidade Estadual do Norte Fluminense (UENF), Campos dos Goytacazes, Rio de Janeiro, Brazil. *P. cincinnata* was donated by the Plantarum Institute, Nova Odessa, São Paulo, Brazil. *P. edulis* was collected in Livramento de Nossa Senhora, Bahia, Brazil.

### 2.2. Genomic DNA Extraction and Next-Generation Sequencing (NGS)

Genomic DNA was extracted according to the protocol proposed by Doyle and Doyle [28]. DNA purification was performed with the addition of 10% sodium acetate (3 M, pH 5.2) and 200% of the final volume of anhydrous ethanol at −20 °C. DNA quantification was performed using the dsDNA BR (Q32850) quantification kit on Qubit^®^ (Thermo Fisher Scientific^®^, Waltham, MA, USA). DNA sample quality was assessed using absorbance ratios of 260/230 and 260/280 in Nanodrop^®^ equipment (Thermo Fisher Scientific^®^, Waltham, MA, USA).

A genomic DNA library of each species was built using Nextera^®^ DNA Sample Preparation (Illumina^®^ref. 15028212, San Diego, CA, USA) with the Nextera^®^ index kit (Illumina^®^ ref. 15028220), following the manufacturer’s recommendations. Fragmentation was performed with 50 ng of the genomic DNA, with purification using the GFX^®^ PCR DNA and the Gel Band Purification KIT (GE^®^ Health Care Life Sciences, Chicago, IL, USA); amplification and linkage of the indexes specific to each library/species and purification were performed using magnetic beads (AMpure XP beads GE^®^ Health Life Sciences).

Genomic libraries had previously been quantified using Qubit^®^dsDNA BR (Q32850). However, the use of the KAPA Library Quantification Kit IlluminaPlatforms (KR0405) was necessary for the final quantification of the pre-sequencing genomic libraries by qPCR. Preparation of qPCR reactions followed the manufacturer’s protocol in ABI Prism Real-Time PCR (Applied Biosystems^®^, Foster City, CA, USA). Qualitative evaluation of the libraries was inferred by analyzing the dissociation curve of the graph obtained after qPCR, where the existence of adapter dimers was evaluated.

Sequencing was carried out at Laboratory of Molecular Markers at the Center of Biotechnology and Genetics (CBG), UESC, Bahia, Brazil. It was conducted on the Illumina^®^Miseq^®^ platform using the MiSeq^®^ Reagent Kit V3 600 cycles (Illumina^®^ref. 15043895) following the manufacturer’s protocol.

### 2.3. Identification and Annotation of Repetitive DNA

A total of 20,261,594, 16,041,900 and 11,493,782 paired-end reads (average read size 300 bp) were obtained for *P. alata*, *P. cincinnata*, and *P. edulis*, respectively. Genome coverage was 2.8× for *P. alata* (1C = 2.156.49 Mbp; [26]), 3.5× for *P. cincinnata* (1C = 1.359.42 Mbp; [25]), and 2.2× for *P. edulis* (1C = 1.545.24 Mbp; [24]). To discover and characterize repetitive sequences, we used the RepeatExplorer pipeline, implemented in the Galaxy server (http://repeatexplorer.org/, accessed on 17 February 2017), which uses genome sequencing data as input and performs graph-based clustering analysis [14,15]. To obtain high-quality reads, we filtered the reads using a cutoff of 30, trimmed them, and filtered them by size (100 bp). Paired-end reads were randomly sampled to cover 5% of the genome of each species: 1,078,246 for *P. alata*, 735,945 for *P. cincinnata*, and 772,620 for *P. edulis*. Reads clustering was performed with a minimum overlap of 55% and a similarity of 90%. Contigs assembled in clusters (CLs) representing repetitions with an abundance >0.01% were considered repetitive DNA. In addition to the use of the RepeatMasker database, available in RepeatExplorer, a custom database was constructed using repetitive sequences of public databases (Repbase, Plant Repeat Database, and National Biotechnology Information Center (NCBI)), totaling 13,648 sequences. After RepeatExplorer analysis, the clusters were examined and annotated manually. After the annotation, the reverse transcriptase (RT) domains of the most abundant LTR retrotransposons were identified using the Protein Domain Search tool [15] and satellite monomers were reconstructed using a Tandem Repeat Analyser—TAREAN [29]. Primers for the RT domains and satellite DNA monomers selected for chromosomal mapping were designed by the Primer3plus tool [30].

### 2.4. Chromosome Mapping of Retrotransposons and satDNA

#### 2.4.1. Probe Preparation

To prepare the probes, the template DNA was amplified using the specific primers designed for RT domain of the selected LTR retrotransposons (Table 1) and the satellite DNA monomers of *P. alata* (PaSat_145 F: ATCGACGTTTCGCTTCAAAT, R: ACGGTGAGGTGCAGTGTTTT) and *P. cincinnata* (PcSat_145 F: GATCCGTGCAAAAGTCACCT, R: GCCGAAACTGGACGAAAAC). The PCR program consisted of 4 min at 94 °C, followed by 30 cycles of 1 min at 94 °C, 1 min at 56 °C, and 2 min at 72 °C, and a final extension for 10 min at 72 °C. After purification, the PCR products for the RT domains of the LTR retrotransposons were labeled with biotin-16-dUTP (Roche Diagnostics^®^, Pleasanton, CA, USA) via PCR. PCR products for satellite DNA of *P. alata* and *P. cincinnata* were labeled with digoxigenin-11-dUTP and biotin-16-dUTP, respectively, via Nick Translation.

#### 2.4.2. Slide Preparation

Roots tips of *P. alata*, *P. cincinnata* and *P. edulis* approximately 1 cm in length were collected and pre-treated with 0.002 M 8-hydroxyquinoline (Merck^®^, Rahway, NJ, USA) for 1 h at room temperature (RT) and 21 h at 8 to 10 °C. After being washed twice in distilled water and fixed in Carnoy II (anhydrous ethanol (Merck^®^, Rahway, NJ, USA)/glacial acetic acid (Merck^®^, Rahway, NJ, USA) [3:1], *v*/*v*) for 3 h at RT, the samples were stored at −20 °C for at least 24 h or until use. For slide preparation, root apices were washed twice in distilled water and incubated with 15 μL of an enzymatic solution containing 2% cellulase (≥0.3 units/mg; Sigma^®^) and 20% pectinase (≥5 units/mg; Sigma^®^, Marlborough, MA, USA) (*w*/*v*) for 80 min at 37 °C. The enzymes were then removed using a micropipette and the roots were washed in distilled water, and then 10 μL of 45% acetic acid (Merck^®^, Rahway, NJ, USA) was added. The roots were macerated using needles under a stereomicroscope, covered with a coverslip, and the cytological material was spread using a thick-tip needle. After spreading, the coverslip was gently pressed with the aid of filter paper to spread the chromosomes, frozen in liquid nitrogen for about 6 min to remove the coverslip, and finally air dried. Slides with good metaphase preparations were maintained at −20 °C until the application of FISH.

#### 2.4.3. Fluorescent In Situ Hybridization (FISH) 

Slide treatment for FISH followed the protocol proposed by Schwarzacher and Heslop-Harrison [31] and Souza et al. [32]. Slides containing cytological preparations with good metaphases were dried at 37 °C for a minimum time of 1 h. The slides were treated with 50 µg/mL RNase (Sigma^®^) in 2×SSC buffer (0.3 M sodium chloride (Sigma^®^); 0.03 M sodium citrate (Sigma^®^)) and incubated in a humid chamber at 37 °C for 1 h. Slides were immersed in 2×SSC at RT twice for 5 min each; 50 μL of 10 mM hydrochloric acid (HCl; Vetec) was added over metaphases for 5 min, and after the removal of HCl, 50 μL of pepsin (Sigma^®^) [10 mg/mL pepsin; 10 mM HCl (1:100 *v*/*v*)] was added and the slides were incubated in a humid chamber for 20 min at 37 °C. The following washing steps were performed on a shaker platform (Biomixer, Mos-1, São Paulo, Brazil) at 120 rpm: the slides were washed 2×SSC at RT twice for 5 min each, immersed in 4% formaldehyde at RT for 10 min, and then washed in 2×SSC twice for 5 min each. Cytological preparations were dehydrated in 70% ethanol and 96% ethanol for 5 min each. After drying the slides at RT for 30 min, the hybridization mixture was added to the final volume of 15 μL containing 50% formamide (Sigma^®^), 10% dextran sulfate (Sigma^®^), 2×SSC, 0.13% SDS (sodium dodecyl sulfate, Bioagency, Hamburg, Germany), and 50 ng of DNA probe. The hybridization mixture was heated at 75 °C for 10 min in a thermocycler (Eppendorf, Mastercycler, Hamburg, Germany) and transferred immediately to ice for 5 min. Slides containing the hybridization mixture were denatured in a thermocycler (Techne, TC-412, Chelmsford, UK) containing a slide adapter at 75 °C for 10 min and incubated in a humid chamber overnight at 37 °C. After hybridization, the slides were immersed in 2×SSC at RT for 5 min to remove the coverslips. The following post-hybridization washes were performed in a water bath (Marconi, MA093/1/E, Piracicaba, Brazil) at 42 °C: two immersions in 2×SSC for 5 min each, two in 0.1×SSC for 5 min each, and two more immersions in 2×SSC for 5 min each. The slides were immersed in a solution with 2% 4×SSC/Tween 20 (Sigma^®^) at RT for 5 min and treated with 50 μL of 5% bovine serum albumin, fraction V (BSA; Sigma^®^). Biotin-16-dUTP-labeled probes were detected with 0.7 μL of fluorescein-isothiocyanate (FITC-Avidin; Vector, Newark, NJ, USA) plus 19.3 μL of 5% BSA per slide. Digoxigenin-11-dUTP-labeled probes were detected with 0.7 μL anti-digoxigenin-rhodamine (Roche™) plus 19.3 μL of 5% BSA per slide. The slides containing the antibodies for detection were incubated in a humid chamber for 1 h at 37 °C. Excess antibodies were removed with three baths in 4×SSC/0.2% Tween20 at RT for 5 min each. The slides were briefly immersed in 2×SSC and the cytological preparations were simultaneously assembled and counterstained with Vectashield^®^ Antifade Mounting Medium with DAPI (H-1200). The slides were stored at 8–10 °C until analysis.

#### 2.4.4. FISH Analysis and Photo-Documentation

Image analysis and photo-documentation were performed using an Olympus BX41 epifluorescence microscope equipped with an Olympus DP25 5MP digital camera and using DP2-BSW software (ver.2.1). In situ hybridizations detected with avidin-FITC were visualized with a U-MWB filter (excitation 450–480 nm/dichroic cutoff 500 nm/emission > 515 nm) and the hybridizations detected with anti-digoxigenin-rhodamine were visualized with a U-MWG filter (excitation 510–550 nm/dichroic cutoff 570 nm/emission > 590 nm). DAPI was visualized with a U-MWU filter (excitation 330–385 nm/dichroic cutoff 400 nm/emission > 420 nm). FITC/DAPI and rhodamine/DAPI overlays were produced with the use of Photoshop SC5 software (ver.12.0).

## 3. Results

### 3.1. Genomic Composition

**Figure 1:**
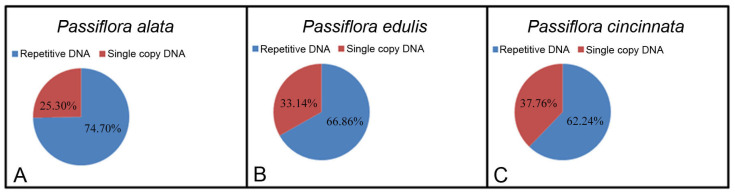
Ratio of repetitive DNA and single-copy sequences in *Passiflora*: (**A**) *P. alata*, (**B**) *P. edulis*, (**C**) *P. cincinnata*.

The characterization of repetitive DNA in *P. alata*, *P. edulis*, and *P. cincinnata* was possible due to sequencing using the Illumina MiSeq platform followed by graph-based clustering via RepeatExplorer. For *P. alata*, 848,168 reads were grouped into 219 clusters (with more than 110 reads), corresponding to 74.70% of the genome (Figure 1A). For *P. edulis*, 551,847 reads were grouped into 250 clusters (with more than 78 reads), corresponding to 66.86% of the genome (Figure 1B). For *P. cincinnata*, 451,899 reads were grouped into 263 clusters (with more than 68 reads), corresponding to 62.24% of the genome (Figure 1C). Although some reads of chloroplast DNA (cpDNA) may have been the result of cpDNA insertions in nuclear plastid DNAs (NUPTs), most cpDNA reads probably resulted from cpDNA contamination and were excluded from the analysis.

In all three species, it was observed that most of the repetitive DNA (abundance > 0.01%) corresponds to the LTR retrotransposons Ty3/Gypsy and Ty1/Copia (Table 2). However, Ty3/Gypsy represented the highest genome proportion in *P. alata* and *P. edulis*, while Ty1/Copia represented the highest genome proportion in *P. cincinnata*. 45S rDNA was represented by two clusters in *P. alata* (CL108 and CL116), four clusters in *P. edulis* (CL108, CL116, CL117, and CL119) and three clusters in *P. cincinnata* (CL70, CL71, and CL130). One cluster in each species corresponded to 5S rRNA: CL175 in *P. alata*, CL229 in *P. edulis*, and CL157 in *P. cincinnata*. One satDNA was identified for each species: CL144 in *P. alata*, CL159 in *P. edulis*, and CL201 and in *P. cincinnata*.

Repetitive DNA types and their genome proportions were identified for each species (Table 2). The total amount of all retrotransposons represented 42.95% of the genome of *P. alata*, 41.25% of *P. edulis*, and 40.34% of *P. cincinnata*. Within the retrotransposons, Ty1/Copia represented 17.05% of *P. alata* genome, 16.70% of *P. edulis* genome, and 24.41% of *P. cincinnata* genome. In turn, Ty3/Gypsy accounted for 25.90% of *P. alata* genome, 24.55% of *P. edulis* genome, and 15.93% of *P. cincinnata* genome. The most abundant Ty1/Copia retrotransposon observed in the three species was the Angela family, representing 9.73% of the genome of *P. alata*, 8.39% of the genome of *P. edulis*, and 13.63% of the genome of *P. cincinnata*. The most abundant Ty3/Gypsy retrotransposon was the Chromovirus family, comprising 15.75% of the genome of *P. alata*, 12.51% of the genome of *P. edulis*, and 7.21% of the genome of *P. cincinnata*. Transposon DNA represented 0.02% of the genome of *P. alata*, 0.62% of the genome of *P. edulis*, and 0.03% of the genome of *P. cincinnata.* 45S and 5S rDNA represented 0.28% and 0.02% of the genome of *P. alata*, 0.62% and 0.01% of the genome of *P. edulis*, and 0.75% and 0.05% of the genome of *P. cincinnata*, respectively. satDNA represented 0.05% of the genome of *P. alata*, 0.04% of the genome of *P. edulis*, and 0.02% of the genome of *P. cincinnata*.

### 3.2. Identification of RT Domains

The most abundant clusters containing the reverse transcriptase (RT) domain were identified by the Protein Domain Search tool and, after identification, the RT domains were isolated within the contigs for chromosome mapping. The selection of the RT domain was based on its high conservation within the retrotransposon polyprotein.

In *P. alata*, among the LTR retrotransposons of the Ty3/Gypsy superfamily that presented in the RT domain, two clusters belonging to the Chromovirus family (CL4 and CL41) and one cluster (CL81) belonging to the Athila family were identified. For the clusters CL41 and CL81, a contig containing the RT domain was found, whereas for CL4, three contigs were identified. Among the retrotransposons of the Ty1/Copia superfamily, one cluster was identified as belonging to the Angela family (CL57) (Figure S1).

In *P. edulis*, among the retrotransposons of the Ty3/Gypsy superfamily that presented in the RT domain, two clusters belonging to the Chromovirus family (CL15 and CL18) and a cluster belonging to the Athila family (CL61) were identified. For the clusters CL15 and CL61, one contig containing the RT domain was found, whereas for CL1, two contigs containing the RT domain were identified. Among the retrotransposons of the Ty1/Copia superfamily, were identified one cluster belonging to the Angela family (CL21) and one cluster belonging to the Maximus/SIRE family (CL82) (Figure S2).

In *P. cincinnata*, among the retrotransposons of the Ty3/Gypsy superfamily that presented in the RT domain, we identified two clusters belonging to the Chromovirus family (CL6 and CL99) and one cluster belonging to the Athila family (CL30). For cluster CL6, we identified three contigs containing the RT domain, whereas only one contig was found for the clusters CL30 and CL99. Among the retrotransposons of the Ty1/Copia superfamily, one cluster belonging to the Maximus/SIRE family (CL55) was identified. For this CL, three contigs containing the RT domain were observed (Figure S3).

### 3.3. Identification and Characterization of Satellite DNA

**Figure 2:**
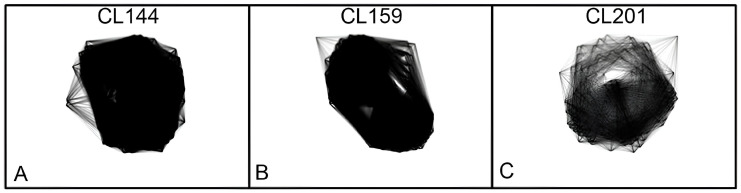
Graphic layouts of the 145 bp satellite DNA clusters identified in *Passiflora*: (**A**) CL144-*P. alata*, (**B**) CL159-*P. edulis*, (**C**) CL201-*P. cincinnata*.

A 145 bp satellite was identified in each species: CL144 in *P. alata* (PaSat_145), CL159 in *P. edulis* (PeSat_145), and CL201 in *P. cincinnata* (PcSat_145). In all three species, a characteristic circular layout of satellite DNA was observed (Figure 2). Using the RepeatExplorer pipeline, satellites that have monomers smaller than the size of the reads used for clustering (100 bp in this analysis) tend to form a circular-spherical graph and those with monomers larger than the read size tend to form a ring-shaped graph [18]. The satellite DNA of *P. edulis* was not used for chromosome mapping in the present work since it had already been mapped in the subtelomeric regions in the chromosomes of *P. edulis* [23].

**Figure 3:**
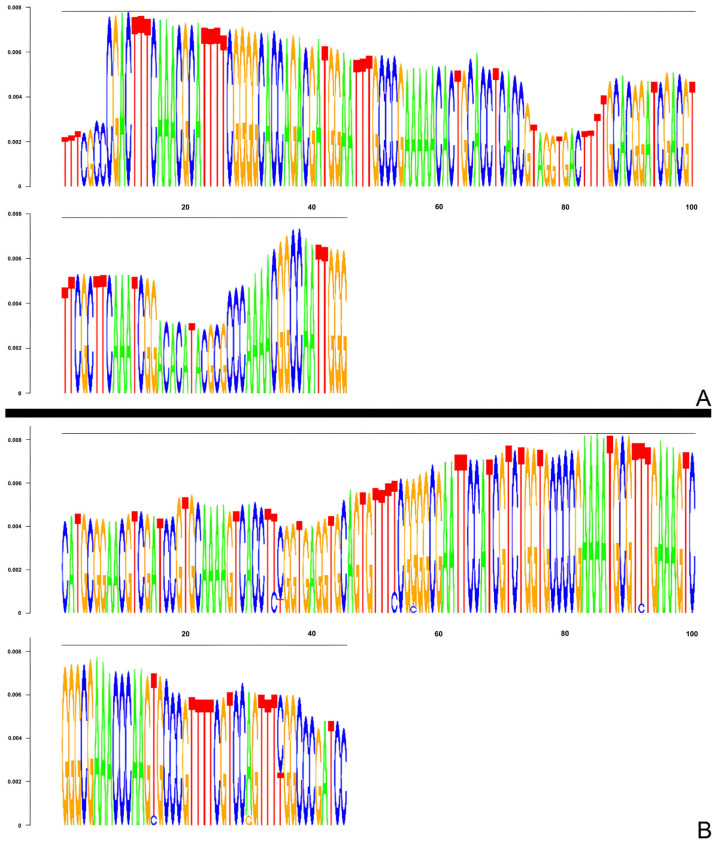
DeBruijn graphs representing the 145 bp consensus monomer of the satellite DNA of *Passiflora* reconstructed using the TAREAN tool: (**A**) *P. alata*, (**B**) *P. cincinnata*.

For *P. alata* and *P. cincinnata*, the sizes and nucleotide sequences of the consensus monomers were reconstructed using the TAREAN tool, which is based on K-mers analysis and represented by the DeBruijn graph (Figure 3). The monomers identified in the two species were blasted against the DNA database (GenBank/NCBI) and no significant similarity was found with the available satellite sequences in the database.

### 3.4. Chromosomal Mapping of Retrotransposons and satDNA

For chromosomal mapping, probes representing the reverse transcriptase (RT) region of each retrotransposon and the selected satDNA monomer were used. Due to the high similarity between the contigs of the same cluster, only one contig was selected for the clusters of retrotransposons with more than one contig with the RT domain. The mapped retrotransposons exhibited a dispersed pattern along most of the chromosomes in the three species; however, the most abundant retrotransposons in the genome proportion showed a greater number of spots of hybridization on the chromosomes. In all three species, the retrotransposons of the Chromovirus family had the highest number of spots of hybridization.

**Figure 4:**
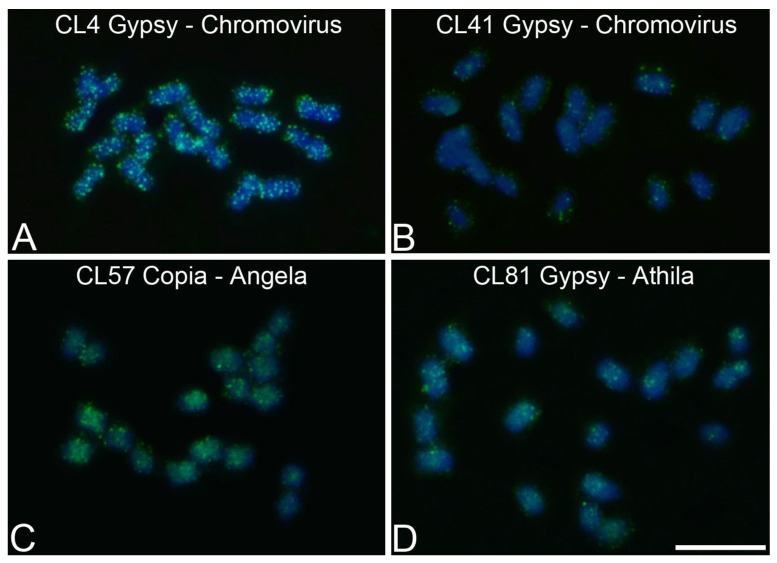
Chromosomal mapping of LTR retrotransposons in metaphase chromosomes of *Passiflora alata* (2*n* = 18) using Fluorescent In Situ Hybridization (FISH): (**A**) CL4 Gypsy/Chromovirus, (**B**) CL41 Gypsy/Chromovirus, (**C**) CL57 Copia/Angela, (**D**) CL81 Gypsy/Athila. Bar = 10 μm.

For *P. alata*, CL4 (Gypsy/Chromovirus) showed the highest abundance of hybridization spots distributed throughout all chromosomes, with intense hybridization signals (Figure 4A), while CL41 (Gypsy/Chromovirus) presented smaller and less intense hybridization spots along the chromosomes (Figure 4B). In CL57 (Copia/Angela), spots of hybridization were observed preferentially in the pericentromeric regions (Figure 4C). CL81 (Gypsy/Athila) presented weak spots of hybridization, which were absent in the arms of some chromosomes (Figure 4D).

**Figure 5:**
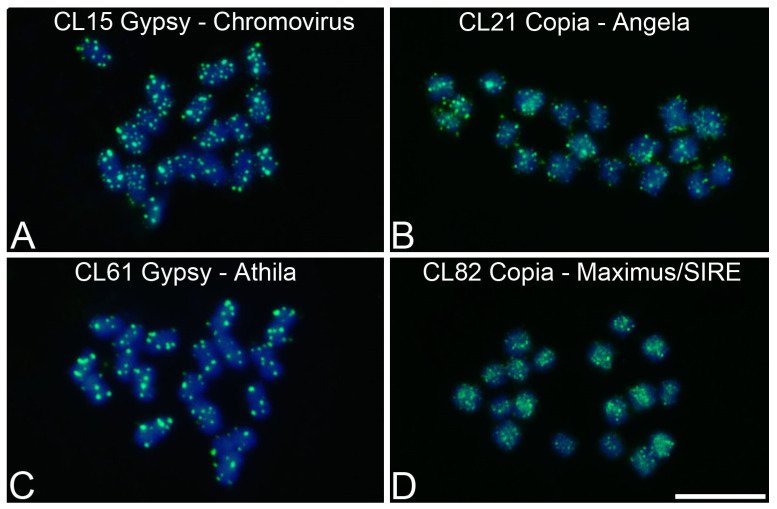
Chromosomal mapping of LTR retrotransposons in metaphase chromosomes of *Passiflora edulis* (2*n* = 18) using Fluorescent In Situ Hybridization (FISH): (**A**) CL15 Gypsy/Chromovirus, (**B**) CL21 Copia/Angela, (**C**) CL61 Gypsy/Athila, (**D**) CL82 Copia/Maximus/SIRE. Bar = 10 μm.

In *P. edulis*, Gypsy/Chromovirus (CL15) showed the highest abundance of spots of hybridization, with isolated and intense signals distributed throughout all chromosomes (Figure 5A). Copia/Angela (CL21) presented a hybridization pattern with greater abundance in some chromosomes compared to others (Figure 5B). Gypsy/Athila (CL61) showed intense, well-defined, and isolated spots of hybridization (Figure 5C). Copia/Maximus/SIRE (CL82) presented spots of hybridization that were more abundant in the proximal regions and less abundant in the telomeric regions (Figure 5D).

**Figure 6:**
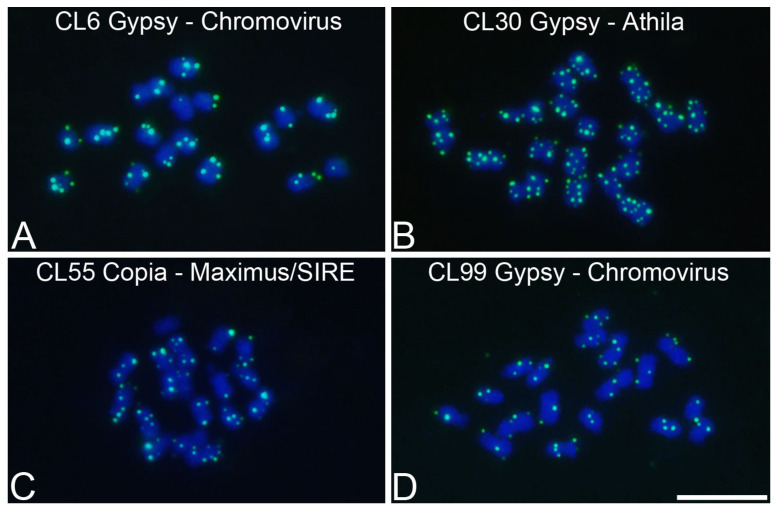
Chromosomal mapping of LTR retrotransposons in metaphase chromosomes of *Passiflora cincinnata* (2*n* = 18) using Fluorescent In Situ Hybridization (FISH): (**A**) CL6 Gypsy/Chromovirus, (**B**) CL30 Gypsy/Athila, (**C**) CL55 Copia/Maximus/SIRE, (**D**) CL99 Gypsy/Chromovirus. Bar = 10 μm.

In *P. cincinnata*, Gypsy/Chromovirus (CL6) showed the brightest spots of hybridization; however, it presented one chromosomal pair with only one weakly labeled spot of hybridization (Figure 6A). Gypsy/Athila (CL30) presented the highest number of spots of hybridization, with well-defined sites (Figure 6B). Copia/Maximus/SIRE (CL55) presented some chromosomes with larger spots of hybridization, some chromosomes with smaller spots of hybridization, and one chromosome with no spots of hybridization (Figure 6C). Gypsy/Chromovirus (CL99) presented the lowest amount of spots of hybridization (Figure 6D).

**Figure 7:**
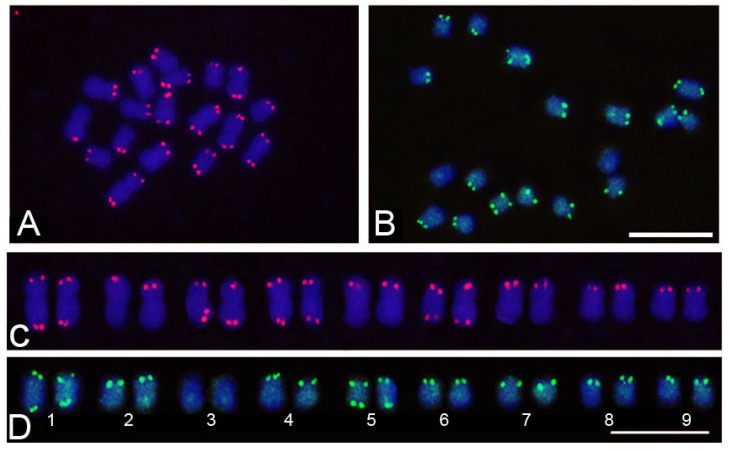
Chromosomal mapping of 145 bp satellite DNA in metaphase chromosomes of *Passiflora* using Fluorescent In Situ Hybridization (FISH): (**A**) CL144 *P. alata* (2*n* = 18), (**B**) CL201 *P. cincinnata* (2*n* = 18), (**C**) cariogram of *P. alata*, (**D**) cariogram of *P. cincinnata*. 1–9 pairs of chromosomes. Bar = 10 μm.

The 145 bp satDNA was mapped to the subtelomeric regions of the chromosomes of *P. alata* and *P. cincinnata*. In *P. alata*, all chromosomes had satellite DNA sites. Four chromosome pairs presented satellite DNA sites in both arms (pairs 1, 3, 4, and 6) and five chromosome pairs presented satellite DNA sites only in the short arm (pairs 2, 5, 7, 8, and 9) (Figure 7A,C). *P. cincinnata* presented two chromosome pairs with satellite DNA sites in both arms (1 and 5), six chromosome pairs with satellite DNA sites in only one arm (2, 4, 6, 7, 8, and 9) and one chromosome pair without the presence of satDNA sites (3) (Figure 7B,D). *P. cincinnata* showed a smaller number of satDNA sites, which is related to its lower proportion in the genome compared to *P. alata*.

## 4. Discussion

The identification of repetitive DNA based on the clustering of reads and their graph representation from low-coverage sequencing has been utilized in a wide variety of plants [18,19]. This approach enables a global characterization of the genome’s repetitive DNA and facilitates comparative analyses within or between genomes [16]. This work presents the first comparative cytogenetic analysis involving species of the genus *Passiflora*. We observed that the largest portion of the genome of the analyzed species is composed of LTR retrotransposons (Gypsy and Copia), as found in other plant species [16,17,20,21]. The highest proportion of LTR retrotransposons, as observed in this study, is a common feature in higher plant genomes, where retrotransposons represent one of the major forces in the evolution of genome size [33,34]. This was also an important evolutionary route in *Passiflora*.

The proportion of repetitive DNA—62.24%, 66.86%, and 74.70% in *P. cincinnata*, *P. edulis*, and *P. alata*, respectively—shows that this type of DNA makes up a significant fraction of their genomes. However, the proportion of repetitive DNA can reach 90% in some plant species with large genomes [35]. The highest proportion of repetitive DNA in *P. alata* genome is possibly related to its larger genome size (1C = 2.156,49 Mbp; [26]), whereas *P. cincinnata* has the smallest proportion of repetitive DNA and the smallest genome size (1C = 1.359,42 Mbp; [25]). In the subgenus *Passiflora*, there is large variation in DNA content (0.26 to 2.68 picograms; 1C) between the species [24,25,26]. The variation in genome size between species with the same chromosome number is generally attributed to variation in the amount of repetitive DNA [27]. Considering that the *Passiflora* species studied present the same chromosomal numbers, our results indicate that the increase in the genome size in *Passiflora* is related to the increase in repetitive DNA, specifically retrotransposons. In the genus *Oryza* (rice), it was observed that two families of Ty3/Gypsy (RIRE2 and Atlantys) are correlated with variation in genome size between different species [36].

The Ty1/Copia superfamily represents the largest proportion of the *P. cincinnata* genome, while the Ty3/Gypsy superfamily represents the highest proportion of the genome of *P. alata* and *P. edulis*. These results show that different retrotransposons can act on the evolution of genome size in *Passiflora*, although the mechanism leading to the preferential amplification of a particular retrotransposon in different taxa is not yet understood [16]. These findings underscore the impact of retrotransposon dynamics on *Passiflora* speciation. In the genus *Helianthus* (sunflower), it has been suggested that the variations observed in the retrotransposons of different species negatively affect chromosomal collinearity, thereby favoring species isolation [20]. Additionally, differences in the ratio between Gypsy and Copia have been observed in different groups. Gypsy was the most abundant retrotransposon in *Helianthus* and *Brachiaria* [37,38], whereas Copia was the most abundant in *Rhynchospora* [39].

*P. cincinnata* is one of the few species of the genus *Passiflora* adapted to the *caatinga* biome in the Brazilian semi-arid region, which is characterized by a low rainfall index. Consequently, this species presents tolerance to drought [40,41]. The difference in repetitive DNA composition between species suggests that their habitats may be related to the abundance of a specific type of retrotransposon. In the genus *Helianthus* (sunflower), it was observed that the retrotransposon Gypsy/Chromovirus is more abundant in perennial species, whereas Copia/Maximus/SIRE is more abundant in annual species, suggesting that their habitats can be related to the abundance of a specific type of repetitive DNA [20,42]. Repetitive DNA elements can impact gene expression and regulation [43] and can be involved in epigenetic modifications, such as DNA methylation and histone modifications, which can regulate gene expression and play a role in habitat adaptation [44].

Sequence variations and quantitative differences between retrotransposons reflect the evolutionary relationships between species. Generally, the quantitative difference is proportional to the divergence between species [17]. In the Musaceae family, the high repetitive DNA similarity within the groups *Musa beccarii*/*M. textilis* and *M. acuminata*/*M. ornata* or *M. acuminata*/*M. ornata*/*M. balbisiana* reflects the phylogenetic relationships between these species [17]. A characterization of repetitive DNA within the phylogenetic context of 23 species of the Fabae tribe revealed significant differences in the extent of sequence conservation between different types of repetitive DNA [19].

Chromosomal mapping showed a dispersed hybridization pattern of retrotransposons in the three species, with no association with a specific chromosomal region. In all three species, Chromovirus family retrotransposons were the most abundant in the chromosomes. Gypsy/Chromovirus retrotransposons represent the oldest and most widely distributed lineage in eukaryotes [45]. A higher proportion of Chromovirus in the genome and, consequently, a greater number of spots of hybridization in the chromosomes may be related to an important role that these retrotransposons play in structuring and organizing the genome of the *Passiflora* species. Although no retrotransposon exclusively associated with the centromeric region has been found, some have been shown to be preferentially associated with pericentromeric regions. Previous studies have shown that pericentromeric retrotransposons are associated with the recruitment of histone-modifying enzymes and with the maintenance of pericentromeric heterochromatin, which is involved in controlling the recombination of centromere regions [46,47].

The proportion of satDNA, which represents 0.05% of the genome of *P. alata*, 0.04% of *P. edulis*, and 0.02% of *P. cincinnata*, in the three analyzed species shows that this repetitive DNA varies between different taxa. In the Musaceae family (banana), the proportion of satellite DNA ranged from 0.005% to 0.592% [17]. In *Silene latifolia*, satellite DNA represented 3.19% of the genome [16]. In *Hippophae rhamnoides* (common sea buckthorn), satellite DNA represented 26.92% of the genome [12]. The atypical abundance of satellite DNA observed in *H. rhamnoides* compared to other plant genomes provides evidence that certain satellites can evolve rapidly in some species [12]. 

Chromosome mapping of 145 bp satDNA in the subtelomeric region of *P. alata* and *P. cincinnata* showed that this satellite DNA is variable among species, presenting differences in the number of sites and in which chromosomal pairs are present. Additionally, it was observed that the number of sites is related to the proportion of satellite DNA in the genome of each species. *P. cincinnata*, which showed the lowest proportion of satellite DNA in the genome, also presented the lowest number of subtelomeric sites, indicating that this repetitive DNA can vary between different species and can be applied in comparative analyses among different species. Similar to *P. edulis* [23], in *P. alata*, 145 bp satDNA sites were observed in all chromosomes, while in *P. cincinnata*, a chromosome pair without 145 bp satDNA was observed, suggesting that this species is the most divergent among these species. Similarly, subtelomeric satellite DNA has already been mapped in several plant species, including *Lycopersicon esculentum* [48], *Nicotiana tabacum* [49], *Oryza sativa* [50], *Zea mays* [51], and *Solanum lycopersicum* [52]. In contrast, the presence of in tandem repeats in distal heterochromatic blocks in *Allium fistulosum* was suggested to move the chiasm away from the ends of the chromosomes during meiosis [53]. In humans, studies of subtelomeric sequences suggest that these regions are extremely dynamic, possibly involved in frequent exchanges of sequences between the ends of non-homologous chromosomes [54].

## 5. Conclusions

Our results showed that an increase in genome size in *Passiflora* species is associated with an increase in repetitive sequences. Regarding the composition of repetitive DNA, *P. cincinnata* exhibited greater divergence compared to *P. alata* and *P. edulis*. Copia was the most abundant in *P. cincinnata*, while Gypsy was the most abundant in *P. alata* and *P. edulis*. Regarding satDNA distribution, *P. cincinnata* has the lowest number of sites and contains a chromosomal pair without satDNA sites. Therefore, retrotransposons and satellite DNA may be useful for comparative analyses between different *Passiflora* species.

## Figures and Tables

**Table 1:** Characterization of the retrotransposons used in the chromosome mapping for *Passiflora alata*, *P. cincinnata*, e *P. edulis*.

Species	Cluster	GP (%) ^1^	Classification	Primers (5′-3′)
*P. alata*	CL4	1.62	Ty3/Gypsy/Chromovirus	F: CTGCAAGACGGTCCTCAACT
				R: GTTCGCTCCGGTTGTGTATT
*P. alata*	CL41	0.73	Ty3/Gypsy/Chromovirus	F: AAATGCGACTCATGCTCCTC
				R: ACCGGTCCTGTTCGTGAAG
*P. alata*	CL57	0.56	Ty1/Copia/Angela	F: TCTCTCGAATCCAGGTTGCT
				R: TCAAGCATGGTCCTTGACAG
*P. alata*	CL81	0.35	Ty3/Gypsy/Athila	F: AGACGGGGATCACGGTTATT
				R:TCTTCTCAATGCAACGCTGT
*P. edulis*	CL15	1.03	Ty3/Gypsy/Chromovirus	F: AGGGAGCAGCAGTATTTTCG
				R: GCTTGTCTCGGAGCGTTT
*P. edulis*	CL21	0.97	Ty1/Copia/Angela	F: ATGGGCCTTAGTTGACTTGC
				R: CATGCAACCATATCTTGACTGA
*P. edulis*	CL61	0.47	Ty3/Gypsy/Athila	F: CCTGTGCAATGTGTTCCAAA
				R: TCCCAATTCAGCACAAGGTT
*P. edulis*	CL82	0.29	Ty1/Copia/Maximus/SIRE	F: GCATACTTTCCTTGTGTGATGAA
				R: GCTTGATGAAAATGGCATGA
*P. cincinnata*	CL6	1.22	Ty3/Gypsy/Chromovirus	F: GCATACTTTCCTTGTGTGATGAA
				R: GCTTGATGAAAATGGCATGA
*P. cincinnata*	CL30	0.73	Ty3/Gypsy/Athila	F: CCAGTGCAGTGTGTTCCAAA
				R: TGCCTTCTCGTACCATGAAAT
*P. cincinnata*	CL55	0.50	Ty1/Copia/Maximus/SIRE	F: TTGGCATCCTCCAAATTGA
				R: GGGAACTTGTTAAAAGGCCTAAA
*P. cincinnata*	CL99	0.17	Ty3/Gypsy/Chromovirus	F: GCTTAGCATAAAGCTTTTCACG
				R: AGCTTAAGCCCATGTGCAGT

^1^ Genome proportion.

**Table 2:** Composition of repetitive DNA in *Passiflora alata*, *P. cincinnata*, and *P. edulis*.

Classification			Genome Proportion (%)		
Repetitive Elements	Superfamily	Family	*P. alata*	*P. edulis*	*P. cincinnata*
LTR Retrotransposons	Ty1/Copia	Angela	9.73	8.39	13.63
		Tork	0.07	0.26	0.32
		Maximus/SIRE	0.07	1.21	2.77
		Bianca	0.00	0.00	0.02
		Unclassified	7.18	6.84	7.67
		Ty1/Copia total	17.05	16.70	24.41
	Ty3/Gypsy	Chromovirus	15.75	12.51	7.29
		Athila	3.11	4.96	4.58
		Unclassified	7.04	7.08	4.06
		Ty3/Gypsy total	25.90	24.55	15.93
Transposon DNA			0.02	0.62	0.03
Pararetrovirus			0.00	0.00	0.01
In tandem	45S DNAr		0.28	0.62	0.75
	5S DNAr		0.02	0.01	0.05
	SatDNAs		0.05	0.04	0.02
Unclassified			31.38	24.32	21.04
Repetitive DNA total			74.70	66.86	62.24
Single copy			25.30	33.14	37.76

## Data Availability

The data presented in this study are available on request from the corresponding author. The data are not publicly available due to privacy restrictions.

## Supplementary Materials

The following supporting information can be downloaded at: https://www.mdpi.com/article/10.3390/genes15040418/s1, Figure S1. Graphic layouts of clusters (CLs) that presented the reverse transcriptase (RT) domain in *Passiflora alata*: (A) CL4 Gypsy/Chromovirus, (B) CL41 Gypsy/Chromovirus, (C) CL57 Copia/Angela, (D) CL81 Gypsy/Athila; Figure S2. Graphic layouts of clusters (CLs) that presented the reverse transcriptase (RT) domain in *Passiflora edulis*: (A) CL15 Gypsy/Chromovirus, (B) CL21 Copia/Angela, (C) CL61 Gypsy/Athila, (D) CL82 Copia/Maximus/SIRE; Figure S3. Graphic layouts of clusters (CLs) that presented the reverse transcriptase (RT) domain in *Passiflora cincinnata*: (A) CL6 Gypsy/Chromovirus, (B) CL30 Gypsy/Athila, (C) CL55 Copia/Maximus/SIRE, (D) CL99 Gypsy/Chromovirus.
