# Supplementary material for: Cytogenomic Characterization of Transposable Elements and Satellite DNA in Passiflora L. Species
Source: Genes (Basel). 2024 Mar 27;15(4):418. doi: 10.3390/genes15040418 (PMC11049143; doi:10.3390/genes15040418)
Supplement: Supplementary file 1 [file genes-15-00418-s001.zip › genes-2914320-supplementary.pdf]

Supplementary material

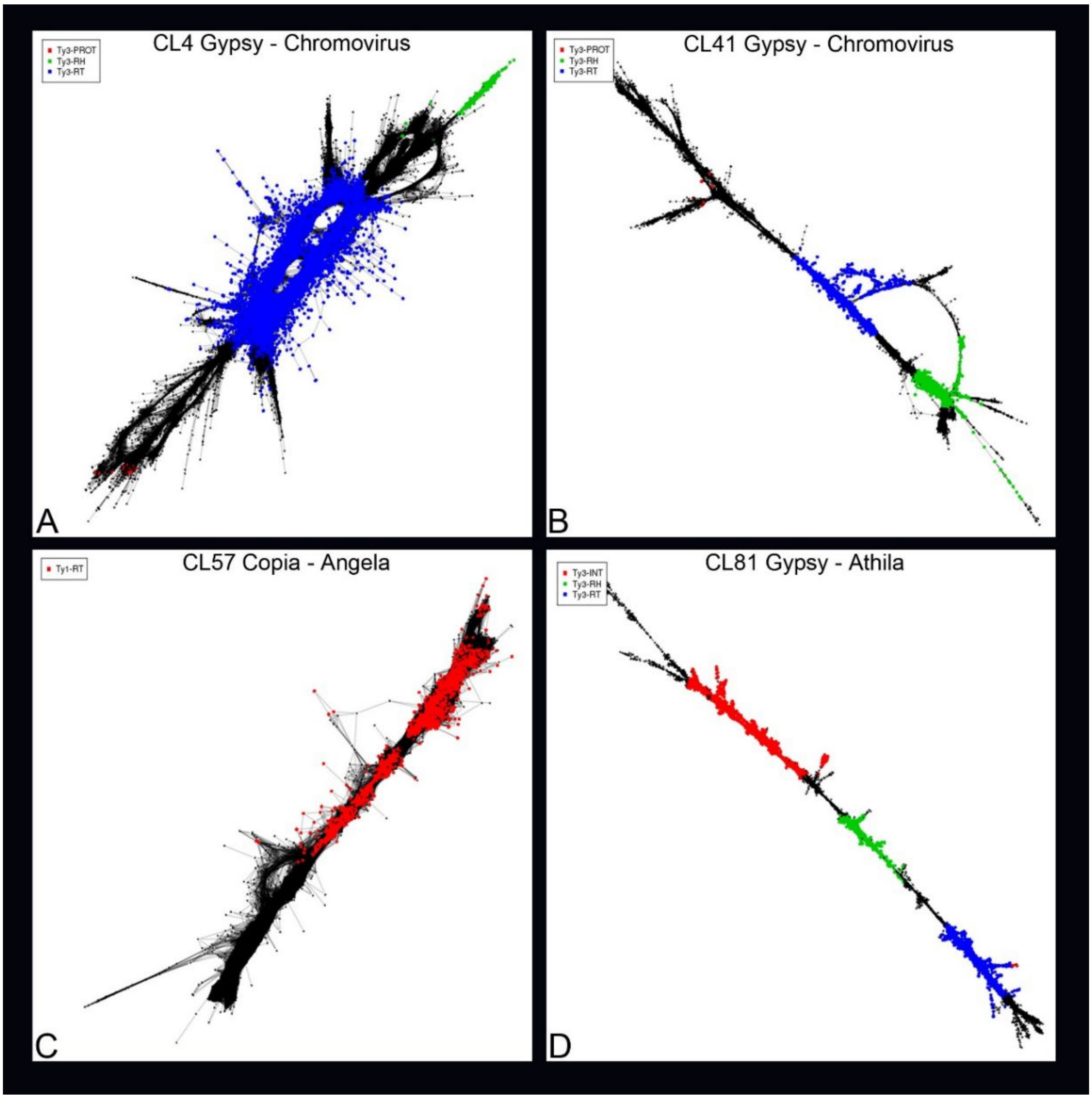

Figure S1. Graphic layouts of clusters (CLs) that presented the reverse transcriptase (RT) domain in *Passiflora alata*: (**A**) CL4 Gypsy/Chromovirus, (**B**) CL41 Gypsy/Chromovirus, (**C**) CL57 Copia/Angela, (**D**) CL81 Gypsy/Athila.

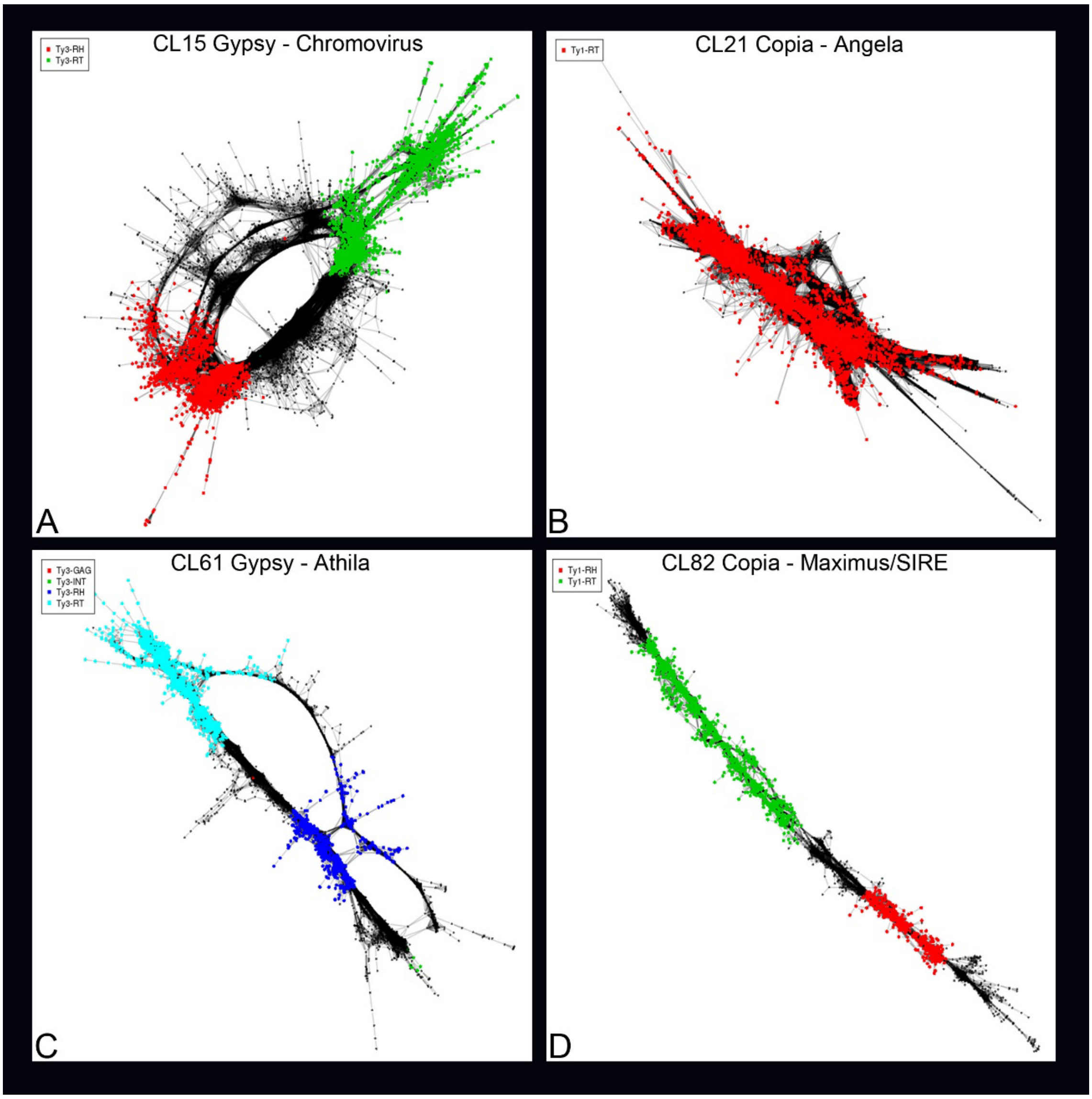

Figure S2. Graphic layouts of clusters (CLs) that presented the reverse transcriptase (RT) domain in *Passiflora edulis*: (**A**) CL15 Gypsy/Chromovirus, (**B**) CL21 Copia/Angela, (**C**) CL61 Gypsy/Athila, (**D**) CL82 Copia/Maximus/SIRE.

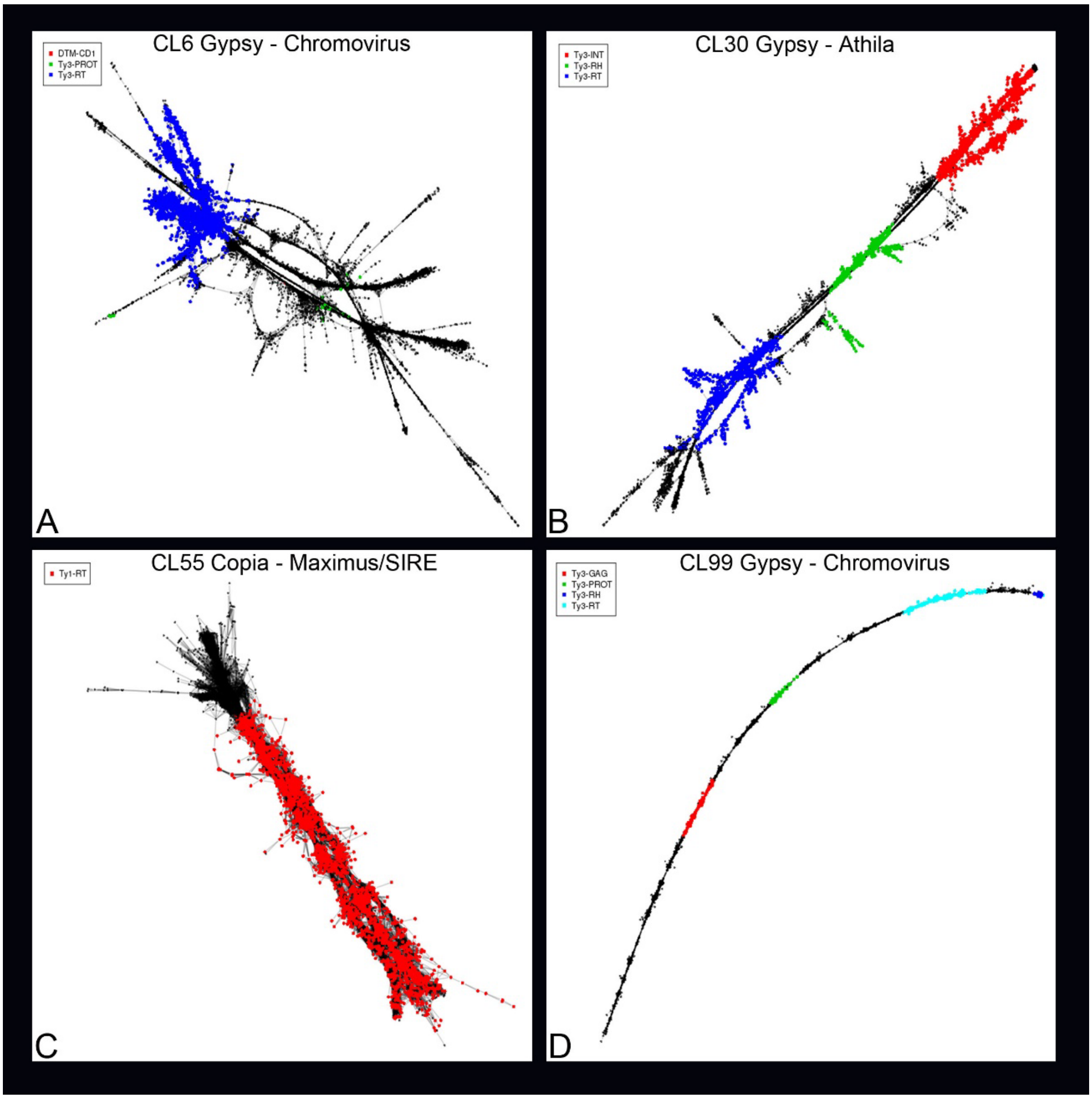

Figure S3. Graphic layouts of clusters (CLs) that presented the reverse transcriptase (RT) domain in *Passiflora cincinnata*: (**A**) CL6 Gypsy/Chromovirus, (**B**) CL30 Gypsy/Athila, (**C**) CL55 Copia/Maximus/SIRE, (**D**) CL99 Gypsy/Chromovirus.
